# Imported malaria in Albania and the risk factors that could allow its reappearance

**DOI:** 10.1186/1475-2875-12-197

**Published:** 2013-06-12

**Authors:** Klodiana Shkurti, Gentian Vyshka, Enkelejda Velo, Arben Boçari, Majlinda Kokici, Dhimitër Kraja

**Affiliations:** 1 Service of Infectious Diseases, University Hospital Centre “Mother Theresa”, Tirana, Albania; 2 Biomedical and Experimental Department, Faculty of Medicine, University of Tirana, Tirana, Albania; 3 Department of Entomology, Institute of Public Health, Tirana, Albania; 4 Faculty of Veterinary Medicine, Agricultural University of Tirana, Tirana, Albania; 5 Biochemical and Microbiological Laboratory, Faculty of Medicine, University of Tirana, Tirana, Albania

**Keywords:** Malaria, *Plasmodium falciparum*, *Plasmodium vivax*, *Plasmodium ovale*, *Anopheles*, Flooding, Migratory movements, Albania

## Abstract

Malaria is an infectious disease gradually becoming a serious concern for public health institutions, even in European countries where the eradication of the disease was previously taken for granted. Albania was listed as an endemic area from the beginning of the 20th Century, but the disease was gradually under control and some decades after the World War II it was merely considered a historical curiosity. Nevertheless, for many reasons, since 1994 and in increasing trend, Albanian health facilities have registered several cases of malaria. Tracing the remnants of the autochthonous disease and finding links with the actual situation seems difficult, due to the relatively long period separating the proclaimed eradication of malaria with the re-appearance of the infection. Among major factors leading to such re-appearance might be massive migratory movements, and environmental changes such as the flooding of areas close to river deltas that flow into the Adriatic and Ionian Seas. These factors, combined with the constant presence of several *Anopheles* species, have led to newly-diagnosed imported malaria cases in Albania. Although all reported cases are considered imported, measures have to be put in place, in order to prevent reappearance of autochthonous malaria cases, and to control disease spread.

## Background

Southern Albania and the western lowland close to the Adriatic seashore have been endemic zones for malaria, and the disease has been a public health concern for changing governments both before and after World War II. The communist regime that ruled the country for almost half a century after World War II invested important, albeit meagre, efforts to eradicate the disease, and programmes to control malaria were in place from 1929 to 1930 [1,2]. Although no reliable source has indicated an incontestable date of its eradication, the last cases were probably treated around the end of 80ies of the last century. There is a scarcity of official data regarding the spread of the disease; the only paper indexed in Medline by an Albanian author, describing the issue of autochthonous malaria in the country, is dated 1962 [3].

The first case of imported malaria, after almost three decades of eradication, was registered in 1994 in a foreign citizen residing in Albania. In 2010 the two first cases of malaria in Albanian nationals, immigrating periodically to Greece, were registered. In 2012, some other five cases were registered, all of them working in Equatorial Guinea, and residing in Vlorë, a southern, seaside city of Albania.

## Presentation of the hypothesis

Sharp outbreaks of re-appearing malaria due to migratory movements, of whatsoever nature, have been registered in other settings [4]. Migratory currents of seasonal workers to Greece and back have been constant on the Albanian-Greek border since the early 1990s, but malaria cases in Albania have been registered only some two decades later. This time delay does not necessarily devalue the ‘imported’ disease hypothesis, and if Greece itself has been free from autochthonous malaria since 1974, the recent findings suggest its re-emergence [5].

Beyond the migratory movements of Albanians consistently southwards, two major factors have to be taken into account: first, the flooding of wide areas of Albanian western lowland and river deltas that flow into the Adriatic Sea; and second, the geographical distribution of *Anopheles* species, whose density in certain areas is sufficient to create *in loco* reservoirs.

In formulating the present hypothesis, authors consider that flooding is not a risk factor of first-hand importance, since none of the seven cases was an inhabitant (even temporarily) of the flooded areas of the Albanian Adriatic coast. Flooding itself has been implicated in the creation of a large number of breeding sites for mosquito-borne diseases and thus favours malaria [6]. However, other authors’ opinions are that dry weather is a risk factor *per se*, because drought conditions can suppress predators of *Anopheles* malaria vectors [7,8].

An important risk factor that is widely formulated and that these findings agree on, are the migratory movements [9]. The geographical distribution of *Anopheles* in Albania is a second major risk factor, which has to be detailed.

## Testing the hypothesis

### *Anopheles* geographical distribution

**Figure 1:**
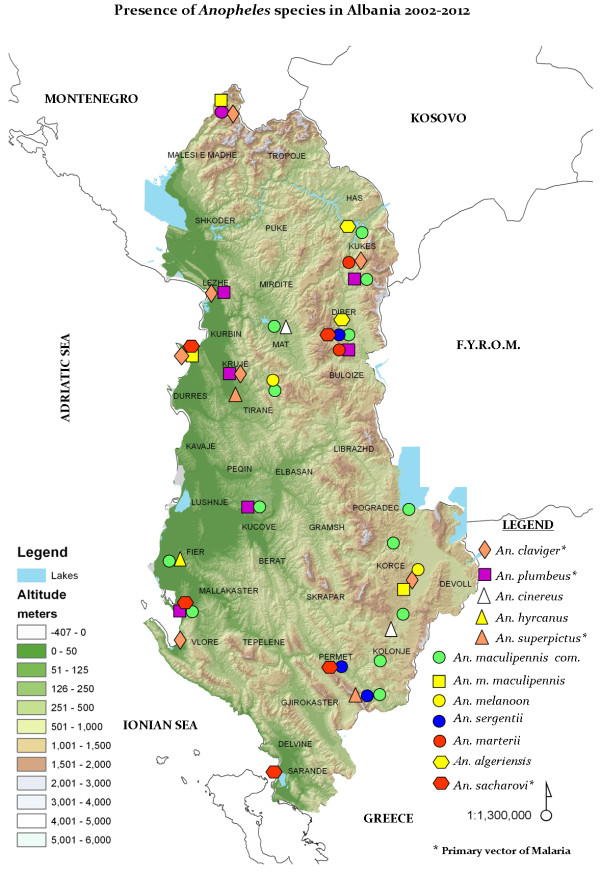
Geographical distribution of Anopheles species in Albania.

The map (Figure 1) shows the presence of *Anopheles* species inside Albania, during the period 2002-2012. The distribution is not uniform, but the presence is significant in southern and south-eastern zones, with *Anopheles sacharovi* being present in two of the main southern border cities, Përmet and Saranda, both important migratory stations, and with *Anopheles claviger* present in Vlorë and in the upland of Devoll, a south-eastern border fluvial area. Worth mentioning is the fact that *An. sacharovi* was considered as the main malaria vector in Albania from World Health Organization (WHO) sources [10].

Note the almost complete absence in the northern area of Shkodër, close to the lake of the same name, bordering Montenegro. Other sources have registered the presence of *Anopheles melanoon* and *Anopheles messeae* in the side of lake in Montenegro, but these were not encountered on the Albanian side of the lake [11,12]. The role of both these species as malaria vectors has been widely contradicted, since they feed mainly on animals [11]. Furthermore, there has been only a single report registering the presence of *An. melanoon,* after long periods of observation in the eastern Mediterranean [13].

The presence of *An. claviger, Anopheles plumbeus, Anopheles superpictus* and *An. sacharovi*, which are considered primary vectors of malaria, can be seen on Figure 1. The geographical distribution of *Anopheles* species seems to follow two major corridors. The first (northwards) starts in the Dibra region and leads toward central Albania, with a strong presence of *Anopheles maculipennis, An. sacharovi* and *An*. *claviger* in areas close to Kosovo and the Former Yugoslavian Republic of Macedonia. The second corridor (southwards) starts on the Albanian-Greek border with a constant presence of *An. maculipennis* and *An*. *claviger* in the Devoll upland, a previously marshy land, where agriculturalization was of major pride to the previous ruling regime. Both corridors follow air currents, with strong winds blowing towards the western lowland and seashores.

### Migratory movements as a human factor

**Figure 2:**
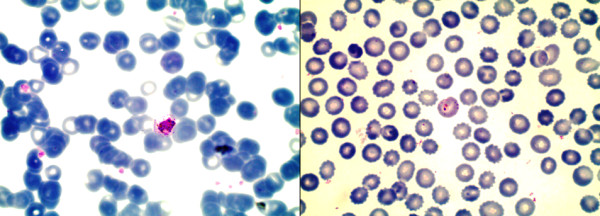
**Peripheral blood stain of two illustrative cases. Left***,* one of two patients infected with *Plasmodium vivax,* with a pro-schizont in the centre of the image. **Right,** blood stain from the only patient infected with *Plasmodium ovale*, showing a young trophozoite including a few stippling. (Giemsa-Romanowski stain, 100 X).

There were two cases of ‘imported’ malaria reported from immigrants working seasonally in Greece (during the year 2010). Both were inhabitants of southern Albanian villages, close to Përmet and Saranda. Five other cases, all of them living in Vlorë, had returned from Equatorial Guinea where they had been working (registered in 2012). There is a strong presence of *An. claviger* in Vlorë (see Figure 1). All seven patients were males of middle age (29-45 years). Of the cases from Equatorial Guinea, four were infected with *Plasmodium falciparum*, and one was infected with *Plasmodium ovale* (see Figure 2). The two individuals returning from Greece showed *Plasmodium vivax*. All cases were registered from the Institute of Public Health in Tirana, and reported to the regional office of WHO. The case characteristics and respective outcomes are summarized in the Table 1.

**Table 1:** Characteristics of seven cases of malaria treated at our facility, 2010-2012

***Type of malaria***	***Age of the patient (years)***	***Sex***	***Diagnostic procedure***	***Country where possibly infected (year of registration)***	***Arrival in Albania to hospitalization (days)***	***Outcome***
*Plasmodium vivax*	37	Male	Blood smear	Greece (2010)	26	Survived
*Plasmodium vivax*	41	Male	Blood smear	Greece (2010)	7	Survived
*Plasmodium falciparum*	29	Male	Blood smear	Equatorial Guinea (2012)	4	Survived
*Plasmodium falciparum*	32	Male	Blood smear	Equatorial Guinea (2012)	4	Survived
*Plasmodium falciparum* (cerebral)	32	Male	Blood smear	Equatorial Guinea (2012)	2	Survived
*Plasmodium falciparum*	45	Male	Blood smear	Equatorial Guinea (2012)	8	Survived
*Plasmodium ovale*	39	Male	Blood smear	Equatorial Guinea (2012)	12	Survived

### Flooding as an environmental risk factor

**Figure 3:**
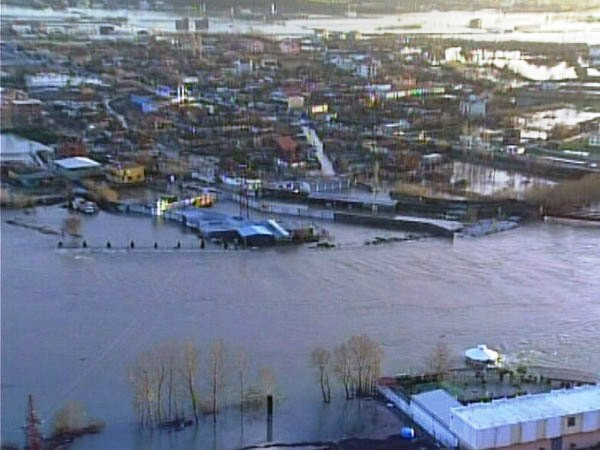
**Flooded area in the Buna river delta which flows to the Adriatic Sea on the northern border of Albania with Montenegro, close to the city of Shkodër.** In the flooded areas of the northern lowland, no presence of *Anopheles* has been registered in Shkodër (see map – Figure 1).

Flooding occurred almost constantly during the period 2008-2010 in the northern lowland close to the Adriatic seashore (see Figure 3). Global warming, deforestation, hydric resource management and other factors have been implicated; nevertheless, there are no cases of malaria, imported or autochthonous, reported from the flooded areas.

## Implications of the hypothesis

It seems that these collected data emphasize the importance of ‘imported’ malaria, since all cases came from other countries. What remains of autochthonous malaria is an enigma, since the two major corridors of *Anopheles’* presence inside Albania line up, one after the other, with the southern Albanian-Greek border. A strong presence of *An. maculipennis* and *An*. *claviger* in the Devoll upland might well be a tolling bell for a (probable) re-appearance of autochthonous malaria, given that more cases of this type of disease have been referred to by Greek authors over the last few years [14,15].

The situation seems to be far from an acute recrudescence. However, if efforts are focused mainly or exclusively on treatment, the burden of disease will gradually increase [16]. Of course, the impact of malaria is much more tragic in other areas, such as sub-Saharan Africa, where about 20% of deaths of children under five years old are attributed to this disease [17].

Controlling the *Anopheles* habitat in the south-eastern border areas of Albania seems a logical measure, given the reported cases of autochthonous malaria in Greece, and the previously marshy area of Devoll upland, close to that same border. Countries with a much harder burden of disease have already proposed and applied control measures, such as larviciding with *Bacillus thuringensis*, by using insect growth regulators, or through the use of a larva-eating fish, *Gambusia affinis,* in targeted zones [18]. All the newly promoted methods will have been preceded by more popular and cheaper forms (insecticides, use of nets, reporting bites and raising awareness). However, insecticides are gradually losing their efficacy and resistance by *Anopheles* to them is a question remaining unresolved [19].

Introducing control measures is becoming necessary at a time of increased migratory movements and geopolitical changes; there are authors that report connections between the collapse of the Soviet Union and epidemics of malaria in neighbouring countries and which clearly underlines the inter-regional effects of disease burden and spread [20].

Among factors analysed above, *Anopheles* geographical distribution is a major risk factor, due to its strong presence in southern and south-eastern areas of Albania, which has to be considered by public health policy makers. The Anopheles species recorded in the present paper (Figure 1) logically fall into the dominant vectors of human malaria, referred from several sources, in the European region [21,22].

On the other hand, there are presently insufficient data regarding mosquito density and respective seasonal frequency in the Albanian regions where the above sketched map suggested a strong presence of *Anopheles*. Countries close to Albania have already put in place epidemiological observation measures, among other even through a strict entomological and vector surveillance [23-25]. The theoretical susceptibility of the main vector species to become infected with *P. vivax*, *P. falciparum* and/or *P. ovale* is another unanswered question for the Albanian region; decades before, it had been considered that exotic strains brought to Europe from Africa were unable to adapt to be transmitted from vectors [26]. Nevertheless, the requested process of adaptation of those strains might have meanwhile progressed, but other parameters, such as the vectorial capacity and susceptibility have to be scrutinized.

The next step in defining the risk of malaria re-emergence in Albania would, therefore, be a thorough study of receptivity, in all suggested parameters (human biting rate, anthropophily, lengths of trophogonic and sporogonic cycles and so on), especially in areas considered at-risk, namely those mapped with a higher presence with *Anopheles* species, and probably the previously marshy uplands of south-eastern Albania, or the regions considered once as endemic for the autochthonous form of the disease [27].

Another challenging factor for clinicians and epidemiologists was the fact that imported cases referred herein had a very different suspected provenance, with two cases imported from Greece and the others from a much more distant country, Equatorial Guinea. This will of course, highlight the impact of a global transportation of imported malaria; such a trend has already been emphasized by others [28].

It seems that history is making its unrelenting return: once Albania was a country of endemic malaria, even becoming a place for laboratory research of mosquitoes [29]. The time for intervening and for replicating the findings of Bates, whose outdoor cage studies in Albania are almost 70 years old, has arrived [11,30].

## Competing interests

The authors declare that they have no competing interests.

## Authors’ contributions

KS and DK are clinicians that diagnosed, cured and followed up all seven malaria patients. GV wrote the manuscript and reviewed the literature. EV sketched the map of *Anopheles* in Albania, and studies their biological behaviour and characteristics. AB offered additional data on the ecology of mosquitoes. MK made the blood stains and other haematologic controls for the group. All authors read and approved the final manuscript.
